# The EUTOS long-term survival (ELTS) score is superior to the Sokal score for predicting survival in chronic myeloid leukemia

**DOI:** 10.1038/s41375-020-0931-9

**Published:** 2020-06-29

**Authors:** Markus Pfirrmann, Richard E. Clark, Witold Prejzner, Michael Lauseker, Michele Baccarani, Susanne Saussele, François Guilhot, Sonja Heibl, Rüdiger Hehlmann, Edgar Faber, Anna Turkina, Gert Ossenkoppele, Martin Höglund, Andrey Zaritskey, Laimonas Griskevicius, Ulla Olsson-Strömberg, Hele Everaus, Perttu Koskenvesa, Boris Labar, Tomasz Sacha, Daniela Zackova, Francisco Cervantes, Adriana Colita, Irena Zupan, Andrija Bogdanovic, Fausto Castagnetti, Joëlle Guilhot, Joerg Hasford, Andreas Hochhaus, Verena S. Hoffmann

**Affiliations:** 1 Institut für Medizinische Informationsverarbeitung, Biometrie und Epidemiologie - IBE, Ludwig-Maximilians Universität, Munich, Germany; 2 Department of Molecular and Clinical Cancer Medicine, University of Liverpool, Liverpool, UK; 3 Medical University of Gdansk, Gdansk, Poland; 4 Clinical Department of Hematology and Oncology L. and A. Seragnoli, S. Orsola-Malpighi Hospital, University of Bologna, Bologna, Italy; 5 III. Medizinische Klinik, Universitätsmedizin Mannheim, Medizinische Fakultät Mannheim der Universität Heidelberg, Mannheim, Germany; 6 Clinical Investigation Centre, INSERM CIC 1402, CHU Poitiers, Poitiers, France; 7 Abteilung für Innere Medizin IV, Klinikum Wels-Grieskirchen, Wels, Austria; 8 ELN Foundation, Weinheim, Germany; 9 Department of Hemato-Oncology, Faculty Hospital Olomouc, Faculty of Medicine and Dentistry, Palacky University, Olomouc, Czech Republic; 10 National Research Center for Hematology, Ministry of Healthcare of the Russian Federation, Moscow, Russian Federation; 11 Department of Hematology, VU University Medical Center, Amsterdam, Netherlands; 12 Institution of Medical Sciences, University of Uppsala, Uppsala, Sweden; 13 Almazov Medical Reseach Centre, Institute of Oncology and Hematology, St Petersburg, Russian Federation; 14 Institute of Clinical Medicine, Vilnius University, Vilnius, Lithuania; 15 Department of Medical Sciences, University of Uppsala and Section of Hematology, Uppsala University Hospital, Uppsala, Sweden; 16 Tartu University Hospital, Tartu, Estonia; 17 Hematology Research Unit Helsinki, University of Helsinki and Department of Hematology, Helsinki University Hospital Comprehensive Cancer Center, Helsinki, Finland; 18 School of Medicine, University of Zagreb, Zagreb, Croatia; 19 Department of Hematology, Jagiellonian Unversity Medical College, Kraków, Poland; 20 Department of Internal Medicine, Hematology and Oncology, University Hospital Brno and Masaryk University, Brno, Czech Republic; 21 Hematology Department, Hospital Clinic, IDIBAPS, Barcelona, Spain; 22 Romanian Academy of Medical Sciences and Medical University, Bucharest, Romania; 23 Department of Hematology, University Medical Centre, Ljubljana, Slovenia; 24 Clinic of Hematology, Clinical Center of Serbia, Faculty of Medicine, University of Belgrade, Belgrade, Serbia; 25 Klinik für Innere Medizin II, Universitätsklinikum Jena, Jena, Germany

**Keywords:** Risk factors, Epidemiology

## Abstract

Prognostic scores support clinicians in selecting risk-adjusted treatments and in comparatively assessing different results. For patients with chronic-phase chronic myeloid leukemia (CML), four baseline prognostic scores are commonly used. Our aim was to compare the prognostic performance of the scores and to arrive at an evidence-based score recommendation. In 2949 patients not involved in any score development, higher hazard ratios and concordance indices in any comparison demonstrated the best discrimination of long-term survival with the ELTS score. In a second step, of 5154 patients analyzed to investigate risk group classification differences, 23% (*n* = 1197) were allocated to high-risk by the Sokal score. Of the 1197 Sokal high-risk patients, 56% were non-high-risk according to the ELTS score and had a significantly more favorable long-term survival prognosis than the 526 high-risk patients according to both scores. The Sokal score identified too many patients as high-risk and relatively few (40%) as low-risk (versus 60% with the ELTS score). Inappropriate risk classification jeopardizes optimal treatment selection. The ELTS score outperformed the Sokal score, the Euro, and the EUTOS score regarding risk group discrimination. The recent recommendation of the European LeukemiaNet for preferred use of the ELTS score was supported with significant statistical evidence.

## Introduction

For patients with Philadelphia chromosome-positive (Ph+) chronic-phase chronic myeloid leukemia (CML), four baseline prognostic scores were addressed by the most recent European LeukemiaNet (ELN) recommendations [1]. First, in 1984, the Sokal score was developed to allocate chemotherapy-treated patients into three risk groups of approximately equal size predicting significantly different overall survival (OS) probabilities [2, 3]. In 1998, the Euro score was proposed to discriminate OS between three risk groups of patients treated with interferon alpha [2, 4]. Using data on patients who were treated with imatinib, in 2011 the European Treatment and Outcome Study for CML (EUTOS) score identified two risk groups with significantly different probabilities of complete cytogenetic response after 18 months of therapy [2, 5], and in 2016, the EUTOS Long-Term Survival (ELTS) score was introduced in order to distinguish three risk groups with significantly different probabilities of dying of CML [6].

Regarding its primary endpoint, the ELTS score was successfully validated in an independent patient sample and showed a superior risk group discrimination compared with the Sokal score [6]. The Sokal score identified 41% of patients as low-risk and 23% as high-risk. The ELTS score, however, identified an absolute proportion of 20% more low-risk patients and 11% fewer high-risk patients [6]. Ten years after the start of first-line imatinib treatment, probabilities of dying of CML were 6 and 8% according to Hehlmann et al. [7] and Molica et al. [8], respectively. These results are rather in line with 12% high-risk patients as suggested by the ELTS score than with 23% high-risk patients as defined by the Sokal score.

The Sokal score has been particularly popular [1]. This may have been due to the preference for risk groups of more equal size, but a more likely reason was lack of acceptance of newer scores. Accordingly, analyses established in major randomized trials continued to be risk stratified by the Sokal score [9–13]. Here, some association between Sokal risk group and clinical outcome was identified [9–11, 13]. While the most recent ELN recommendations advise risk assessment with the ELTS score [1], it is hence still essential to provide convincing data-based evidence when arguing for its preference over others.

The aim of this work was to compare the prognostic discrimination between the Sokal score [3] and the ELTS score [6] and to provide an evidence-based recommendation of which score to apply. Although the focus was on the comparison between the enduringly popular Sokal score and the relatively new ELTS score, results for the Euro and the EUTOS score are also provided.

## Patients and methods

### Patients

In 2007, a registry of CML patients was established by the ELN and maintained within the EUTOS framework [5]. This registry contains individual data on adult patients who were prospectively enrolled between 2002 and 2006, either within or outwith a clinical trial (in-study and out-study sections, respectively) [5, 14]. Further patient eligibility criteria for both registry sections were diagnosis of Ph+ and/or BCR-ABL1-positive CML in chronic phase, no transcript type other than b2a2 and/or b3a2, and any form of imatinib-based treatment within 6 months from diagnosis [5, 14]. In accordance with these criteria, 2205 patients with data on all variables of each score were retrieved from the in-study section [6]. While data on the in-study patients remained unchanged for the present report, follow-up was updated in 2016 for most patients in the out-study section. Two of the 1120 cases reported earlier [6] were identified as double data entries and were left out from further analyses. A third population-based component of the registry accumulated data on adult patients newly diagnosed between 2008 and 2013 [15]. Apart from adulthood, Ph+ and/or BCR-ABL1-positive CML was the only inclusion criterion [15]. For the population-based section, the same inclusion criteria were chosen as for the two other sections, except that the restriction on patients with first-line imatinib treatment within 6 months from diagnosis was relaxed. Of the 1831 patients finally included, 68 had received first-line dasatinib (4%) and 247 (14%) first-line nilotinib treatment; similarly for 78 patients (4%), treatment start was later than 6 months after diagnosis. Relaxation of the two criteria was based on the observation that both had no association with survival probabilities in the population-based section.

At first, the score comparisons were based on the 2949 patients with data entirely independent of any score development. In a second step, data of the in-study sample used for the development of the ELTS score were added. Only after addition of these patients was the number of events sufficient in order to assess the adequacy of low- or high-risk categorization between the different scores.

### Definitions and endpoints

OS time was calculated from the start date of tyrosine kinase inhibitor (TKI) treatment to death or to the latest follow-up date. Progression-free survival time was calculated like survival time but ended with the observation of progression. Progression was defined by the observation of accelerated phase or blast crisis, with both phases determined according to the ELN criteria [16]. Chronic phase was defined by the absence of progression [16]. Only death after recorded disease progression was regarded as “death due to CML”. Death without prior disease progression was rated as “death unrelated to CML”. For details regarding the calculation of the Sokal [3], the ELTS [6], the Euro [4], and the EUTOS score [5], see Supplementary Table 1.

### Statistical analysis

OS probabilities were calculated by the Kaplan–Meier method, and the hazards ratios (HRs) for dying from any cause were calculated by the Cox regression model [17]. When differentiating competing causes of death, cumulative incidence probabilities of dying of CML were obtained using the Aalen–Johansen estimator [18, 19] and the subdistribution hazards ratios (SHRs) for dying of CML were obtained using the Fine–Gray model [20]. Like the Aalen–Johansen estimator, the Fine–Gray model and its SHRs consider death unrelated to CML as the competing event to death due to CML, the event of interest. Both the hazards from the Cox model as well as the SHRs were compared by the Wald test. To assess discrimination of prognostic models, concordance probabilities were estimated using the truncated concordance index suggested by Wolbers et al. [21]. For the description of discrimination ability over time, the truncation times 1, 5, and 10 years were considered. A higher concordance index hints at a better discrimination of the survival outcome. With indices greater than 50, a prognostic model provides clinically useful information different from chance; the closer to 100, the more supportive the model is.

Lauseker and Zu Eulenburg elucidated that the use of the competing risk model leads to biased cumulative incidence probability estimates when the censoring mechanism differs between status, e.g., between patients in chronic- or progressive- phase [22]. In the case of a status-dependent censoring mechanism, they showed that the progressive illness-death model should be preferred over the competing risk model (see Supplementary Fig. 1 for a comparison of the models). Accordingly, in the presence of status-dependent censoring, the ability to discriminate probabilities of dying of CML was additionally investigated with the progressive illness-death model. For this, the associations between risk group and transition probabilities were considered [23].

For the two-sided *P* values, the unadjusted significance level of 0.05 was applied for all statistical tests. Estimates were presented with 95% confidence interval (95% CI). More on statistical methods is given in the Supplementary appendix.

## Results

### Prognostic discrimination in 2949 patients from the combined out-study and population-based sections

For the out-study data, origin and details as well as the validation of a significant discrimination of the probabilities of dying of CML by the ELTS score have been previously described [6, 14].

Adding the population-based section to the 1118 out-study patients, a validation sample of 2949 patients independent of any score development was achieved. The combined sample consisted of 52% males. Median age of the 2949 patients was 52 years (range: 18–91 years) and median follow-up was 3.3 years (range: 0.01–12.6 years). Altogether, 236 patients died, of whom 89 (38%) died of CML. Six-year OS probability in the 2949 patients was 88% (95% CI: 86–89%), and 6-year probability of death due to CML was 5% (95% CI: 4–6%).

#### Prognostic discrimination of cumulative incidence probabilities of dying of CML

**Fig. 1:**
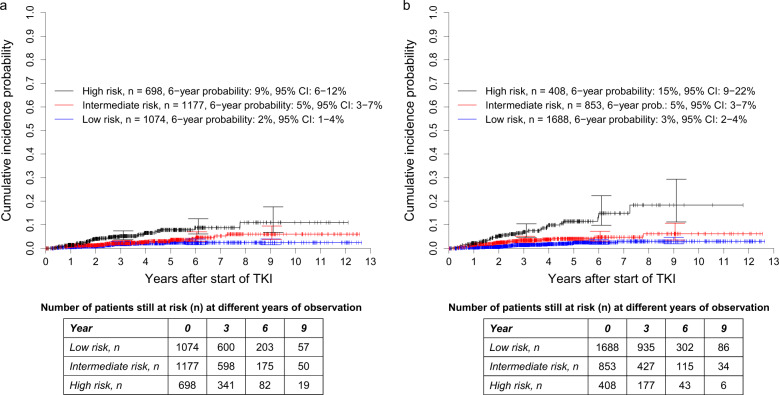
Cumulative incidence probabilities of dying because of CML in 2949 patients from the combined out-study and population-based registry sections. **a** Stratified for the risk groups according to the Sokal score and **b** Stratified for the risk groups according to the ELTS score. At 3, 6, and 9 years, horizontal crossbars indicate the upper and lower limit of the 95% confidence interval (CI) for the estimated probability. **a** The high-risk group of the Sokal score, though not the intermediate-risk group, had significantly higher probabilities of dying because of CML than the low-risk group, *P* < 0.0001 and *P* = 0.0835, respectively. The corresponding SHRs were 3.559 (95% CI: 2.030–6.240) and 1.668 (95% CI: 0.934–2.978). The concordance indices at 1, 5, and 10 years were 59.7, 62.4, and 63.3, respectively. **b** The intermediate- and high-risk groups of the ELTS score had significantly higher probabilities of dying due to CML than the low-risk group with *P* = 0.0031 and *P* < 0.0001, respectively. The corresponding hazard ratios were 2.203 (95% CI: 1.306–3.718) and 5.646 (95% CI: 3.397–9.387). The concordance indices at 1, 5, and 10 years were 68.0, 66.0, and 68.1, respectively.

The high-risk group of the Sokal score (*n* = 698, 24%), though not the intermediate-risk group (*n* = 1177, 40%), had significantly higher probabilities of dying because of CML than the low-risk group (*n* = 1074, 36%), *P* < 0.0001 and *P* = 0.0835, respectively (Fig. 1a). The corresponding SHRs were 3.559 (95% CI: 2.030–6.240) and 1.668 (95% CI: 0.934–2.978). The concordance indices at 1, 5, and 10 years were 59.7, 62.4, and 63.3, respectively.

With the ELTS score, both the intermediate- (*n* = 853, 29%; *P* = 0.0031) and the high-risk group (*n* = 408, 14%; *P* < 0.0001) had significantly higher probabilities of dying because of CML than the low-risk group (*n* = 1688, 57%, Fig. 1b). The corresponding SHRs were 2.203 (95% CI: 1.306–3.718) and 5.646 (95% CI: 3.397–9.387). The concordance indices at 1, 5, and 10 years were 68.0, 66.0, and 68.1. Discrimination abilities were worse with the Euro and the EUTOS score (Supplementary Fig. 2a–b). The Euro score was not able to find a significant discrimination between the intermediate- and the low-risk group, and the EUTOS score was not able to find a significant discrimination between the low- and the high-risk group.

#### State-dependent censoring: application of the progressive illness-death model

In the combined out-study/population-based sample, 153 patients (5%) experienced progression. The cumulative hazard of censoring was significantly higher for patients in progressive phase (*P* < 0.0001). Differences in the state occupation probabilities for death after progression were observed (Supplementary Fig. 3). After 8 years, the probability of death after progression was 7.3% with the progressive illness-death model and 5.7% with the competing risk model. In contrast, for death without progression probability differences were small (10.5 and 10.6%).

The estimated associations between risk group and transition probabilities in the progressive illness-death model are shown in Supplementary Table 2. Compared with the ELTS score, none of the three other prognostic models displayed a better discrimination of transition probabilities (Supplementary Table 2).

#### Prognostic discrimination of OS probabilities

**Fig. 2:**
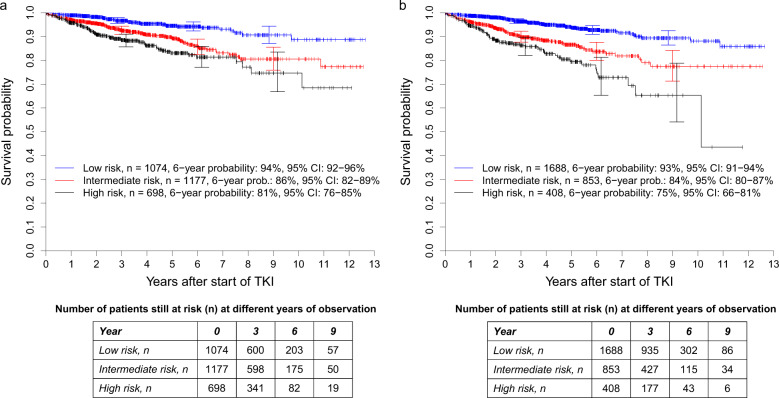
Overall survival probabilities in 2949 patients from the combined out-study and population-based registry sections. **a** Stratified for the risk groups according to the Sokal score and **b** Stratified for the risk groups according to the ELTS score. At 3, 6, and 9 years, horizontal crossbars indicate the upper and lower limit of the 95%-confidence interval (CI) for the estimated probability. **a** The intermediate- and high-risk groups of the Sokal score had significantly lower survival probabilities than the low-risk group with both *P* < 0.0001. The corresponding hazard ratios were 2.256 (95% CI: 1.590–3.201) and 3.384 (95% CI: 2.359–4.852). The concordance indices at 1, 5, and 10 years were 62.9, 62.0, and 61.3, respectively. **b** The intermediate- and high-risk groups of the ELTS score had significantly lower survival probabilities than the low-risk group with both *P* < 0.0001. The corresponding hazard ratios were 2.479 (95% CI: 1.836–3.345) and 4.012 (95% CI: 2.884–5.582). The concordance indices at 1, 5, and 10 years were 65.6, 64.0, and 64.0, respectively.

The intermediate- (HR: 2.256 [95% CI: 1.590–3.201] and high-risk groups (HR: 3.384 [95% CI: 2.359–4.852] of the Sokal score had significantly lower survival probabilities than the low-risk group with both *P* < 0.0001 (Fig. 2a). The concordance indices at 1, 5, and 10 years were 62.9, 62.0, and 61.3, respectively.

With slightly higher hazard ratios and concordance indices of 65.6, 64.0, and 64.0 at 1, 5, and 10 years, the same was observed for the intermediate- (HR: 2.479 [95% CI: 1.836–3.345] and high-risk groups (HR: 4.012 [95% CI: 2.884–5.582] of the ELTS score (Fig. 2b).

While the HRs and the concordances indices of the Euro score were slightly less favorable than the ELTS score, the EUTOS score failed to discriminate risk groups (Supplementary Fig. 4a–b).

### Prognostic discrimination in 5154 patients from all three combined registry sections

The sample of all three combined registry sections consisted of 5154 patients with 52% males and a median age of 52 years (range: 18–91 years). With a median follow-up of 5.3 years (range: 0.01–12.6 years), 429 deaths were recorded, 175 (41%) of which were due to CML. Six-year survival probability of all patients was 90% (95% CI: 89–81%) and 6-year probability of death due to CML was 4% (95% CI: 4–5%).

#### Prognostic discrimination of cumulative incidence probabilities of dying of CML

**Fig. 3:**
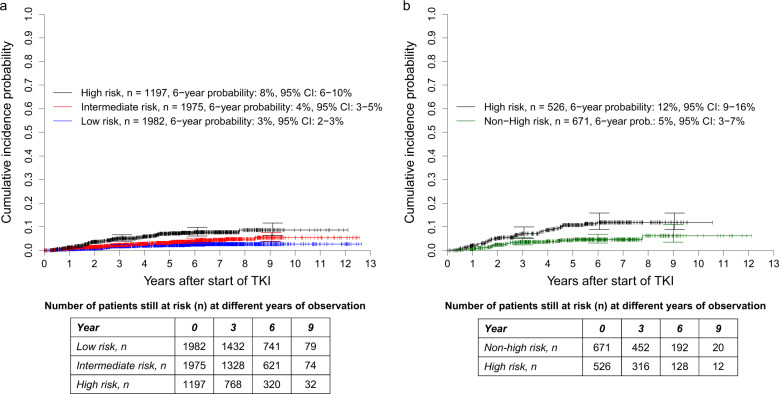
Cumulative incidence probabilities of dying due to CML in 5154 patients from the combined in-study, out-study, and population-based registry sections. **a** Stratified for the risk groups according to the Sokal score and **b** with the 1197 high-risk patients according to the Sokal score stratified for non-high-risk and high-risk according to the ELTS Score. At 3, 6, and 9 years, horizontal crossbars indicate the upper and lower limit of the 95% confidence interval (CI) for the estimated probability. **a** The intermediate- and high-risk groups of the Sokal score had significantly higher probabilities of dying due to CML than the low-risk group with *P* = 0.0088 and *P* < 0.0001, respectively. The corresponding hazard ratios were 1.695 (95% CI: 1.142–2.515) and 3.161 (95% CI: 2.146–4.655). The concordance indices at 1, 5, and 10 years were 58.8, 62.1, and 62.2, respectively. **b** The high-risk group according to both scores had significantly higher probabilities of dying due to CML than the non-high-risk group identified by the ELTS score, *P* = 0.0003. The corresponding hazard ratio was 0.415 (95% CI: 0.256–0.671). The concordance indices at 1, 5, and 10 years were 63.3, 60.8, and 59.9, respectively.

The intermediate- (*n* = 1975, 38%) and the high-risk groups of the Sokal score (*n* = 1197, 23%) had significantly higher cumulative incidence probabilities of dying due to CML than the low-risk group (*n* = 1982, 38%), *P* = 0.0088 and *P* < 0.0001, respectively (Fig. 3a). The corresponding SHRs were 1.695 (95% CI: 1.142–2.515) and 3.161 (95% CI: 2.146–4.655). The concordance indices at 1, 5, and 10 years were 58.8, 62.1, and 62.2.

Of the 1197 patients allocated to high-risk by the Sokal score, 671 (56%) were classified as non-high-risk by the ELTS score. Compared with the 526 patients identified as high-risk by both scores, the cumulative incidence probabilities of dying because of CML were significantly lower for the 671 ELTS non-high-risk patients (SHR: 0.415 [95% CI: 0.256–0.671], *P* = 0.0003, Fig. 3b). The concordance indices at 1, 5, and 10 years were 63.3, 60.8, and 59.9, respectively.

**Fig. 4:**
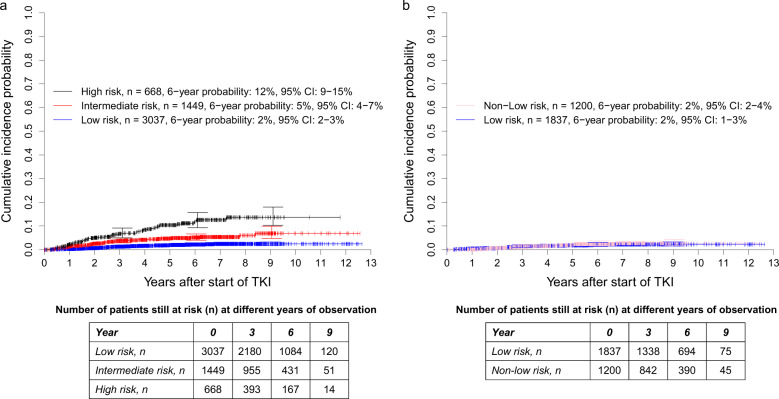
Cumulative incidence probabilities of dying due to CML in 5154 patients from the combined in-study, out-study, and population-based registry sections. **a** Stratified for the risk groups according to the ELTS score and **b** with the 3037 low-risk patients according to the ELTS score stratified for low-risk and non-low-risk according to the Sokal Score. At 3, 6, and 9 years, horizontal crossbars indicate the upper and lower limit of the 95% confidence interval (CI) for the estimated probability. **a** The intermediate- and high-risk groups of the ELTS score had significantly higher probabilities of dying due to CML than the low-risk group with both *P* < 0.0001. The corresponding hazard ratios were 2.584 (95% CI: 1.795–3.721) and 5.667 (95% CI: 3.912–8.209). The concordance indices at 1, 5, and 10 years were 69.6, 66.8, and 67.3, respectively. **b** The non-low-risk group identified by the Sokal score had no significantly different probabilities of dying due to CML than the low-risk group according to both scores, *P* = 0.6635. The corresponding hazard ratio was 1.129 (95% CI: 0.653–1.951). The concordance indices at 1, 5, and 10 years were not analysable, 47.6, and 47.7, respectively.

Compared with the low-risk group (*n* = 3037, 59%), the cumulative incidence probabilities of dying because of CML were significantly higher in the intermediate- (*n* = 1449, 28%, SHR: 2.584 [95% CI: 1.795–3.721]) and the high-risk groups (*n* = 668, 13% SHR: 5.667 [95% CI: 3.912–8.209]) of the ELTS score, with both *P* < 0.0001 (Fig. 4a). The concordance indices at 1, 5, and 10 years were 69.6, 66.8, and 67.3, respectively.

Of the 3037 patients identified as low-risk by the ELTS score, the Sokal score allocated 1200 (40%) to non-low-risk. In relation to the low-risk patients, the cumulative incidence probabilities of dying of CML of the 1200 Sokal non-low-risk patients were hardly different (SHR: 1.129 [95% CI: 0.653–1.951], *P* = 0.6635, Fig. 4b).

With reference to its low-risk group, the Euro score identified significantly higher cumulative incidence probabilities of dying because of CML in high-risk patients (*P* < 0.0001) but failed to do so in patients with intermediate risk (*P* = 0.3768, Supplementary Fig. 5a). The EUTOS score found significantly higher cumulative incidence probabilities of dying in high-risk patients (*P* = 0.0002, Supplementary Fig. 5b).

#### No state-dependent censoring in the 5154 patients from all three combined registry sections

In the patient sample made up of data from all three registry sections, 275 patients had disease progression (5%). The cumulative hazard of censoring was not significantly different between the phases (*P* = 0.2868) and differences in the state occupation probabilities between the statistical models were not of any relevance (Supplementary Fig. 6).

#### Prognostic discrimination of OS probabilities

**Fig. 5:**
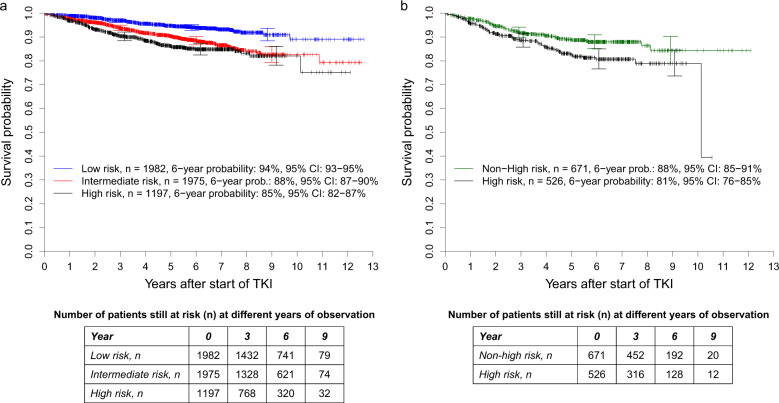
Overall survival probabilities in 5154 patients from the combined in-study, out-study, and population-based registry sections. **a** Stratified for the risk groups according to the Sokal score and **b** with the 1197 high-risk patients according to the Sokal score stratified for non-high-risk and high-risk according to the ELTS Score. At 3, 6, and 9 years, horizontal crossbars indicate the upper and lower limit of the 95%-confidence interval (CI) for the estimated probability. **a** The intermediate- and high-risk groups of the Sokal score had significantly lower survival probabilities than the low-risk group with both *P* < 0.0001. The corresponding hazard ratios were 2.049 (95% CI: 1.607–2.611) and 2.596 (95% CI: 2.009–3.355). The concordance indices at 1, 5, and 10 years were 61.2, 60.6, and 59.7, respectively. **b** The high-risk group according to both scores had significantly lower survival probabilities than the non-high-risk group identified by the ELTS score, *P* = 0.0041. The hazard ratio for non-high- to high-risk patients was 0.615 (95% CI: 0.442–0.857). The concordance indices at 1, 5, and 10 years were 56.5, 55.6, and 55.4, respectively.

The intermediate- (HR: 2.049 [95% CI: 1.607–2.611]) and high-risk groups (HR: 2.596 [95% CI: 2.009–3.355]) of the Sokal score had significantly lower survival probabilities than the low-risk group, with both *P* < 0.0001 (Fig. 5a). The concordance indices at 1, 5, and 10 years were 61.2, 60.6, and 59.7.

The 526 high-risk patients according to both scores had significantly lower survival probabilities than the 671 non-high-risk patients identified by the ELTS score (*P* = 0.0041, Fig. 5b). The HR for non-high- to high-risk patients was 0.615 (95% CI: 0.442–0.857); concordance indices at 1, 5, and 10 years were 56.5, 55.6, and 55.4

**Fig. 6:**
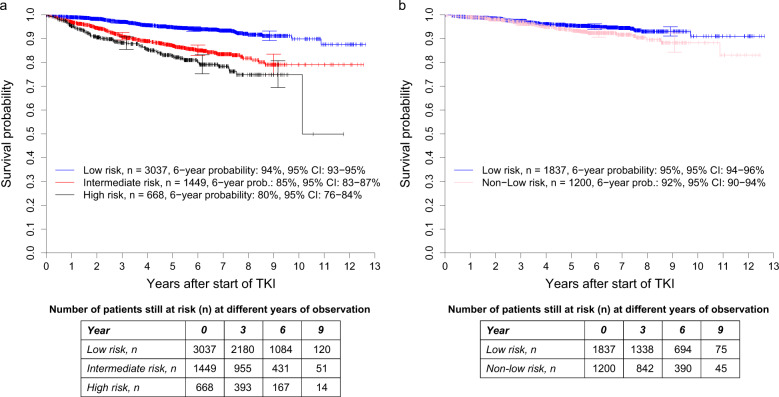
Overall survival probabilities in 5154 patients from the combined in-study, out-study, and population-based registry sections. **a** Stratified for the risk groups according to the ELTS score and **b** with the 3037 low-risk patients according to the ELTS score stratified for low-risk and non-low-risk according to the Sokal Score. At 3, 6, and 9 years, horizontal crossbars indicate the upper and lower limit of the 95% confidence interval (CI) for the estimated probability. **a** The intermediate- and high-risk groups of the ELTS score had significantly lower survival probabilities than the low-risk group with both *P* < 0.0001. The corresponding hazard ratios were 2.631 (95% CI: 2.116–3.273) and 3.675 (95% CI: 2.861–4.720). The concordance indices at 1, 5, and 10 years were 66.6, 63.8, and 63.7, respectively. **b** The non-low-risk group identified by the Sokal score had significantly lower survival probabilities than the low-risk group according to both scores, *P* = 0.0147. The corresponding hazard ratio was 1.490 (95% CI: 1.082–2.053). The concordance indices at 1, 5, and 10 years were 49.8, 54.4, and 54.4, respectively.

With reference to the low-risk group of the ELTS score, the HRs of the intermediate- and high-risk groups were 2.631 (95% CI: 2.116–3.273) and 3.675 (95% CI: 2.861–4.720), respectively (both *P* < 0.0001, Fig. 6a) and the concordance indices at 1, 5, and 10 years were 66.6, 63.8, and 63.7.

The 1200 non-low-risk patients identified by the Sokal score had significantly lower survival probabilities than the 1837 low-risk patients according to both scores (*P* = 0.0147, Fig. 6b). The corresponding hazard ratio was 1.490 (95% CI: 1.082–2.053). The concordance indices at 1, 5, and 10 years were 49.8, 54.4, and 54.4, respectively.

Like the Sokal and ELTS scores, the Euro score suggested an intermediate- and a high-risk group with significantly lower OS probabilities compared with low-risk patients (both *P* < 0.0001, Supplementary Fig. 7a) while the EUTOS score failed to discriminate significantly different OS probabilities (*P* = 0.0739, Supplementary Fig. 7b).

## Discussion

Although first described over 30 years ago, the Sokal score remains popular for risk group discrimination, despite suggesting that, at diagnosis, more than 20% of chronic-phase patients are at high-risk with respect to OS—even in the presence of TKIs—and despite the availability of the ELTS score developed in imatinib-treated patients [6]. The main objective of this work is to provide evidence-based information on what score should be preferred, comparing prognostic discrimination performance between the Sokal and the ELTS score.

To pay tribute to the improved survival evoked by TKI therapy, when developing the ELTS score, the focus was the probabilities of dying of CML (i.e., after progression) rather than dying of any cause. In 2949 patients independent of any score development, unlike the ELTS score, the Sokal score failed to recognize significantly different cumulative incidence probabilities of dying of CML between intermediate- and low-risk patients. Secondly, in relation to the low-risk group, the SHRs as well as the concordance indices were always higher with the ELTS score indicating a better discrimination than with the Sokal score. This result was also observed in the combined sample of 5154 patients from all three registries.

A limitation of the prognostic discrimination comparisons in the combined out-study/population-based sample of 2949 patients was the probable state-dependent censoring. This led to slightly biased cumulative incidence probabilities for death after progression when compared with the gold standard of the progressive illness-death model. Applying the illness-death model, the significantly different hazards for the transitions into progression and into death in chronic phase confirmed a satisfactory discrimination between the risk groups of the ELTS score (Supplementary Table 2). No other score provided a better discrimination of risk groups.

In the samples of 2949 and 5154 patients, for both the Sokal and the ELTS score, all pairwise risk group comparisons led to significant differences in OS probabilities. Again, in any case, the HRs as well as the concordance indices were always higher with the ELTS score than with the Sokal score.

While the sample of 2949 independent patients guaranteed an unbiased comparison between the Sokal and the ELTS score, the sample of 5154 patients was needed for provision of event numbers high enough to investigate risk group classification differences between the scores. Of 1197 patients allocated to high-risk by the Sokal score, the ELTS score classified 56% as non-high-risk. Compared with the 526 high-risk patients according to both scores, the cumulative incidences of dying of CML were significantly lower and OS probabilities were significantly higher for the 671 ELTS non-high-risk patients. For 56% of 1197 patients the allocation of high-risk by the Sokal score was inappropriate. Of 3037 patients identified as low-risk by the ELTS score, the Sokal score classified 1200 (40%) as non-low-risk. The cumulative incidences of dying of CML were only slightly different from those of the remaining 1837 low-risk patients, pointing to another inappropriate classification by the Sokal score. However, in relation to the 1837 patients assessed as low-risk by both scores, OS was significantly lower. Nevertheless, at 92% after 6 years (95% CI: 90–94%) and 88% after 9 years (95% CI: 84–92%), OS probabilities were still high for the 1200 Sokal non-low-risk patients.

HRs and concordance indices showed the best prognostic discrimination in the unbiased comparisons in the 2949 independent patients. Since the ELTS score was developed in the 2205 in-study patients, their inclusion in the total sample of 5154 patients meant some advantage for the ELTS score compared with the other scores. The extent of this limitation cannot be quantified, but in consideration of the very distinctive results, it is still fair to conclude that the risk of inappropriate classification is decidedly higher with the Sokal score.

Successful validation of the ELTS score and superiority in comparison with other scores were also reported by Geelen et al [24]. In 709 patients with first-line imatinib treatment, only the ELTS score was able to identify three pairwise significantly different risk groups with respect to OS and to achievement of a first major molecular response. With only 23 deaths after progression, the ELTS score also provided satisfactory differences in cumulative incidences of death due to CML, but numbers were too low to allow a reliable assessment of prognostic performance. Molica et al. compared the four prognostic systems in 459 individuals treated with imatinib as first-line TKI [8]. Of 51 deaths, only ten were assessed as CML related. The authors judged that the ELTS score predicted probabilities of death better than the other scores. While Yang et al. observed the most distinct risk group discrimination of OS probabilities with the ELTS score in 462 imatinib-treated Chinese patients with a median follow-up of 69 months [25], Millot et al. found that its three risk groups differed significantly from each other with respect to progression-free survival in 350 children with imatinib as first-line treatment—despite only 23 events (progression or death) [26]. In both studies, the authors concluded that the ELTS score outperformed all other scores [25, 26]. However, instead of the conventional Sokal score, Millot et al. considered the Sokal score for younger patients (≤45 years) [26, 27].

In 202 Italian patients ≥65 years treated with imatinib or nilotinib, in contrast to the Sokal score, the ELTS score provided significant discrimination of the three risk groups regarding major (BCR-ABL1 ≤ 0.1%, international scale, IS) and deep molecular remission (BCR-ABL1 ≤ 0.01%, IS) and the probabilities of leukemia-related deaths [28]. The ELTS score also worked best when applied to 258 patients diagnosed in advanced phase [29]. Lauseker et al. concluded that the ELTS score could be applied to distinguish long-term survival between high-risk and non-high-risk patients until a better model developed in patients with accelerated phase and/or blast crisis is introduced [29].

The ELTS score has been validated several times for its ability to significantly discriminate risk groups regarding long-term survival outcome but mainly in patients first-line treated with imatinib [6, 8, 15, 24–26, 28]. Despite significantly faster achievement of molecular reponses with second generation TKIs [10, 13, 30–33], first-line treatment with imatinib and its generics is still widespread. Most physicians continue to see room for first-line treatment with imatinib depending on age, comorbidities, kinase domain mutations, treatment goal, costs, and availability of generic imatinib [1, 33–37]. In prognostic support of first-line treatment selection, the ELTS score offers the most appropriate risk group classification. This is also of interest as imatinib has fewer side effects than second generation TKIs, and it is perceived that a statistically significant overall superiority in long-term efficacy over imatinib has not yet been shown for another TKI [1, 33, 36, 37]. There is indication that the ELTS score would also discriminate risk groups with respect to long-term survival if a second generation TKI were chosen as first-line treatment [24]. More evidence is needed. A large patient sample would be necessary to recognize significant differences in long-term survival between TKIs within a certain risk group.

Regarding risk group discrimination, the ELTS score outperformed the Sokal score, the Euro, and the EUTOS score. Due to our large patient sample, it was possible to show, for the first time with statistical significance, that the Sokal score is much more likely to provide an incorrect risk group classification. The mechanism behind the superiority of the ELTS score is its development in imatinib-treated patients and its different weighting of the four prognostic factors, together with a more adequate patient distribution into risk groups (about 60%/30%/10%) than the Sokal score (about 40%/40%/20%) in times when patients have much better survival prospects due to TKIs.

In the most recently published ELN recommendations, the panel recommend the use of the ELTS score as the preferred method to assess baseline CML risk. Through our work, we back the ELN recommendation with statistical evidence. A valid score and its common application support comparative assessment of efficacy and safety. The ELTS score can be calculated via the “Hematology app” or the website: https://www.leukemia-net.org/content/leukemias/cml/elts_score.

### Supplementary information

Supplementary Information

## Data Availability

For original data, please contact markus.pfirrmann@ibe.med.uni-muenchen.de. Deidentified individual participant data are available upon request and agreement of the scientific committee and the data security officer of our faculty.

## Code availability

Most analyses were undertaken using SAS (version 9.4). The truncated concordance index was calculated using the function *pec* implemented in the programming software R (version 3.4.3) [38]. Estimates of the competing risk and the progressive illness-death model were obtained from the R function *etm* and the association between risk group and transition probabilities was assessed using the R function *mstate* [23, 39].
