# Supplementary material for: The EUTOS long-term survival (ELTS) score is superior to the Sokal score for predicting survival in chronic myeloid leukemia
Source: Leukemia. 2020 Jun 29;34(8):2138–49. doi: 10.1038/s41375-020-0931-9 (PMC7387299; doi:10.1038/s41375-020-0931-9)
Supplement: Supplementary file 1 — Supplementary Information [file 41375_2020_931_MOESM1_ESM.docx]

**SUPPLEMENTARY INFORMATION**

**Table of contents**

SUPPLEMENTARY METHODS – Page 2

SUPPLEMENTARY RESULTS – Page 3

SUPPLEMENTARY REFERENCES – Page 5

SUPPLEMENTARY TABLES – Page 6

SUPPLEMENTARY FIGURES – Page 10

**SUPPLEMENTARY METHODS**

**Survival time in the case of allogeneic hematopoietic stem cell transplantation**

Any survival endpoint was censored at the time of allogeneic hematopoietic stem cell transplantation (HSCT) in first chronic phase; due to transplant-related mortality, survival probabilities after HSCT in the first chronic phase were considered not to be representative for survival probabilities had TKI treatment been continued. With the limited survival prospects after progression, survival endpoints were not censored at the time of allogeneic HSCT in advanced phase when TKI treatment had already failed.

**Competing risks**

The Fine-Gray model differentiates between the event of interest (e.g. death due to CML) and competing events (death unrelated to CML).(1) The Fine-Gray model takes into account that patients experiencing the competing event are not at risk for the event of interest, anymore. Probabilities of dying due to CML do not range over the whole probability distribution between 0 and 100%, if competing events are present. Probabilities of dying due to CML only range over a “subdistribution”, e.g. from 0 to 80%, if 20% competing events are observed. This is considered by the subdistribution hazards provided by the Fine-Gray model.

**Truncated concordance index**

Referring to the truncated concordance index suggested by Wolbers et al.(2), the inverse probability of censoring weighted estimates of concordance were based on the marginal Kaplan-Meier estimator for the censoring distribution of the respective dataset, i.e. either the out-study registry plus the population-based registry or all three combined registry sections.

**Comparison of the estimates between the competing risk model and the progressive illness-death model**

In contrast to the competing risk model, the progressive illness-death model incorporates the transition from chronic phase to progression (Supplementary Figure 1) and is thus considered the gold standard.(3) However, since the competing risk model is easier to understand and implemented in statistical software packages like SAS, it was preferred in case of status-independent censoring. To examine the presence of status-dependent censoring, for each status, cumulative hazards of censoring were calculated with the Nelson-Aalen estimator and compared by the Wald test which was applied to the hazard ratio gained from time-dependent Cox regression.(4)

**SUPPLEMENTARY RESULTS**

**Investigating status-independent censoring in the different patient samples**

The population-based registry strived for the inclusion of any CML patient of a certain region.(5) Lauseker und Zu Eulenburg surmised that with the progression of CML, patients were inclined to change from a local hematologist to a specialized university hospital and thus, the hazard of loss to follow-up was increased as compared with patients staying in chronic phase. As a consequence, compared with the progressive illness-death model, the competing risk model underestimated the probabilities of dying of CML in the population-based registry.(3) The out-study registry could have been affected by the same problem; also here the cumulative hazard of censoring was significantly higher for patients in progressive phase (P = 0.0118).

The estimated associations between risk group and transition probabilities in the progressive illness-death model in 2949 patients from the combined out-study and population-based registry sections are shown in Supplementary Table 2. When compared with the low-risk group of the ELTS score, the hazards for transition into progression were significantly higher in the intermediate- (HR: 2.158 [95% CI: 1.466-3.176], P = 0.0001) and the high-risk group (HR: 4.784 [95% CI: 3.236-7.074], P <  0.0001). The same holds for the transition hazards into death in chronic phase, with the respective HR of 2.601 [95% CI: 1.804-3.750], P < 0.0001) in the intermediate- and of 3.061 [95% CI: 1.961-4779], P < 0.0001) in the high-risk group. No significant differences between any risk groups were observed with respect to the transitions from progression to death after progression.

The significantly different hazards for the transitions into progression and into death in chronic phase confirmed a satisfactory discrimination between the risk groups of the ELTS score (Supplementary Table 2). Once in progression, the hazards for transition into death after progression were not significantly different. This is not contradictory to the findings in the competing risk model. Not considering the transition from chronic to progressive phase, the competing risk model provides increasing hazards to experience progression and subsequent death in one go. In contrast, the progressive illness-death model disentangles the two states. However, with both models, the higher the risk group, the higher the proportion of patients who died in progression. No other score provided a better discrimination of risk groups than the ELTS score, also when using the illness-death model (Supplementary Table 2).

The in-study registry consisted of randomized clinical trials where data is more often collected and checked for completeness. A significantly higher cumulative hazard of censoring for patients in chronic phase suggested a rather particular focus on the follow-up of patients with disease progression (P = 0.0438). However, this had hardly any effect on the state occupation probabilities for death after progression. After 8 years, the probability of death after progression was 4.0% with the progressive illness-death model and 4.2% with the competing risk model. With about half of the events in the in-study registry, the cumulative hazards of censoring and the state occupation probabilities were levelling out when data on all three registries were combined in one patient sample (Supplementary Figure 6).

**SUPPLEMENTARY REFERENCES**

1. Fine JP, Gray RJ. A Proportional Hazards Model for the Subdistribution of a Competing Risk. J Am Stat Assoc. 1999;94:496-509.

2. Wolbers M, Blanche P, Koller MT, Witteman JC, Gerds TA. Concordance for prognostic models with competing risks. Biostatistics. 2014;15:526-39.

3. Lauseker M, Zu Eulenburg C. Analysis of cause of death: Competing risks or progressive illness-death model? Biom. J. 2019;61:264-74.

4. Therneau TM, Grambsch, PM. Modeling Survival Data: Extending the Cox Model. Springer: New York, USA, 2000.

5. Hoffmann VS, Baccarani M, Hasford J, Lindoerfer D, Burgstaller S, Sertic D, et al. The EUTOS population-based registry: incidence and clinical characteristics of 2904 CML patients in 20 European Countries. Leukemia. 2015;29:1336-43.

**SUPPLEMENTARY TABLES**

**Table 1.** Established baseline prognostic scores in chronic myeloid leukemia.

|  | *Formula* | *Risk group* |
| --- | --- | --- |
| *Sokal score^a^* | Sokal score = exp ( 0.0116 x (age [in years] – 43.4)  + 0.0345 x (spleen size [cm below costal margin] – 7.51)  + 0.1880 x ((platelet count [in 10^9^/L] /700)² – 0.563)  + 0.0887 x (blasts [% in peripheral blood]– 2.10) ) | Low risk: <0.80  Intermediate risk:  ≥0.80 and ≤1.20  High risk: >1.20 |
| *Euro score^b^* | Euro score = ( 0.6666 x age [0 when age < 50 years; 1, otherwise]  + 0.0420 x spleen size [cm below costal margin]  + 0.0584 x blasts [% in peripheral blood]  + 0.0413 x eosinophils [%in peripheral blood]  + 0.2039 x basophils [0 when basophils [% in peripheral blood] < 3; 1, otherwise]  + 1.0956 x platelet count [0 when platelets count [in 10^9^/L]  < 1500; 1, otherwise] ) x 1000 | Low risk: ≤780  Intermediate risk:  >780 and ≤1480  High risk: >1480 |
| *EUTOS score^c^* | EUTOS score = 7 x basophils [% in peripheral blood]  + 4 x spleen size [cm below costal margin] | Low risk: ≤87  High risk: >87 |
| *ELTS score^d^* | ELTS score = 0.0025 x (age [in years] / 10)^3^  + 0.0615 x spleen size [cm below costal margin]  + 0.1052 x blasts [% in peripheral blood]  + 0.4104 x (platelet count [in 10^9^/L] / 1000)^-0.5^ | Low risk: ≤1.5680  Intermediate risk:  >1.5680 and ≤2.2185  High risk: >2.2185 |

*^a^*Sokal JE, Cox EB, Baccarani M, Tura S, Gomez GA, Robertson JE, et al. Prognostic discrimination in "good-risk" chronic granulocytic leukemia. Blood. 1984;63:789-799.

*^b^*Hasford J, Pfirrmann M, Hehlmann R, Allan NC, Baccarani M, Kluin-Nelemans JC, et al. A new prognostic score for survival of patients with chronic myeloid leukemia treated with interferon alfa. Writing Committee for the Collaborative CML Prognostic Factors Project Group. J Natl Cancer Inst. 1998;90:850-858.

*^c^*Hasford J, Baccarani M, Hoffmann V, Guilhot J, Saussele S, Rosti G, et al. Predicting complete cytogenetic response and subsequent progression-free survival in 2060 patients with CML on imatinib treatment: the EUTOS score. Blood. 2011;118:686-692.

*^d^*Pfirrmann M, Baccarani M, Saussele S, Guilhot J, Cervantes F, Ossenkoppele G, et al. Prognosis of long-term survival considering disease-specific death in patients with chronic myeloid leukemia. Leukemia. 2016;30:48-56.

**Table 2.** Associations between risk groups according to the prognostic scores and transition probabilities in the progressive illness-death model in 2949 patients from the combined out-study and population-based registry sections

| **ELTS score** | | | | | | |
| --- | --- | --- | --- | --- | --- | --- |
| Hazard ratios of intermediate- to low-risk group with respect to the transition probabilities | | | | | | |
| *Transitions* | *Hazard ratio* | | *Lower 95% confidence limit for hazard ratio* | *Upper 95% confidence limit for hazard ratio* | *P* | |
| *From chronic phase to progression* | 2.158 | | 1.466 | 3.176 | 0.0001 | |
| *From chronic phase to death w/o progression* | 2.601 | | 1.804 | 3.750 | <0.0001 | |
| *From progression to death after progression* | 1.638 | | 0.955 | 2.812 | 0.0733 | |
| Hazard ratios of high- to low-risk group with respect to the transition probabilities | | | | | |  |
| *Transitions* | *Hazard ratio* | *Lower 95% confidence limit for hazard ratio* | | *Upper 95% confidence limit for hazard ratio* | *P* |  |
| *From chronic phase to progression* | 4.784 | 3.236 | | 7.074 | <0.0001 |  |
| *From chronic phase to death w/o progression* | 3.061 | 1.961 | | 4.779 | <0.0001 |  |
| *From progression to death after progression* | 1.458 | 0.873 | | 2.436 | 0.1500 |  |

| **Sokal score** | | | | | | |
| --- | --- | --- | --- | --- | --- | --- |
| Hazard ratios of intermediate- to low-risk group with respect to the transition probabilities | | | | | | |
| *Transitions* | *Hazard ratio* | | *Lower 95% confidence limit for hazard ratio* | *Upper 95% confidence limit for hazard ratio* | *P* | |
| *From chronic phase to progression* | 1.803 | | 1.166 | 2.789 | 0.0081 | |
| *From chronic phase to death w/o progression* | 2.633 | | 1.691 | 4.099 | <0.0001 | |
| *From progression to death after progression* | 1.522 | | 0.829 | 2.797 | 0.1755 | |
| Hazard ratios of high- to low-risk group with respect to the transition probabilities | | | | | |  |
| *Transitions* | *Hazard ratio* | *Lower 95% confidence limit for hazard ratio* | | *Upper 95% confidence limit for hazard ratio* | *P* |  |
| *From chronic phase to progression* | 3.542 | 2.305 | | 5.441 | <0.0001 |  |
| *From chronic phase to death w/o progression* | 3.201 | 1.996 | | 5.134 | <0.0001 |  |
| *From progression to death after progression* | 1.383 | 0.786 | | 2.434 | 0.2609 |  |
| **Euro score** | | | | | | |
| Hazard ratios of intermediate- to low-risk group with respect to the transition probabilities | | | | | | |
| *Transitions* | *Hazard ratio* | | *Lower 95% confidence limit for hazard ratio* | *Upper 95% confidence limit for hazard ratio* | *P* | |
| *From chronic phase to progression* | 1.388 | | 0.948 | 2.033 | 0.0923 | |
| *From chronic phase to death w/o progression* | 3.066 | | 2.004 | 4.691 | <0.0001 | |
| *From progression to death after progression* | 1.643 | | 0.968 | 2.788 | 0.0658 | |
| Hazard ratios of high- to low-risk group with respect to the transition probabilities | | | | | |  |
| *Transitions* | *Hazard ratio* | *Lower 95% confidence limit for hazard ratio* | | *Upper 95% confidence limit for hazard ratio* | *P* |  |
| *From chronic phase to progression* | 3.786 | 2.454 | | 5.840 | <0.0001 |  |
| *From chronic phase to death w/o progression* | 3.064 | 1.718 | | 5.466 | 0.0002 |  |
| *From progression to death after progression* | 1.664 | 0.922 | | 3.002 | 0.0907 |  |
| **EUTOS score** | | | | | | |
| Hazard ratios of high- to low-risk group with respect to the transition probabilities | | | | | |  |
| *Transitions* | *Hazard ratio* | *Lower 95% confidence limit for hazard ratio* | | *Upper 95% confidence limit for hazard ratio* | *P* |  |
| *From chronic phase to progression* | 2.103 | 1.436 | | 3.079 | 0.0001 |  |
| *From chronic phase to death w/o progression* | 1.055 | 0.644 | | 1.729 | 0.8306 |  |
| *From progression to death after progression* | 0.581 | 0.331 | | 1.022 | 0.0594 |  |

**SUPPLEMENTARY FIGURES**

**Figure 1. The progressive illness-death model and the competing risks model applied to occupation states in chronic myeloid leukemia**

Progression

Death after progression

CML, chronic phase

Death without progression

Red lines: Competing risk model

Black lines: Progressive illness-death model

Figure according to Lauseker and Zu Eulenburg(3)^(FIG1)^

**Figure 2. Cumulative incidence probabilities of dying because of CML in 2949 patients from the combined out-study and population-based registry sections**

**a) stratified for the risk groups according to the Euro score**


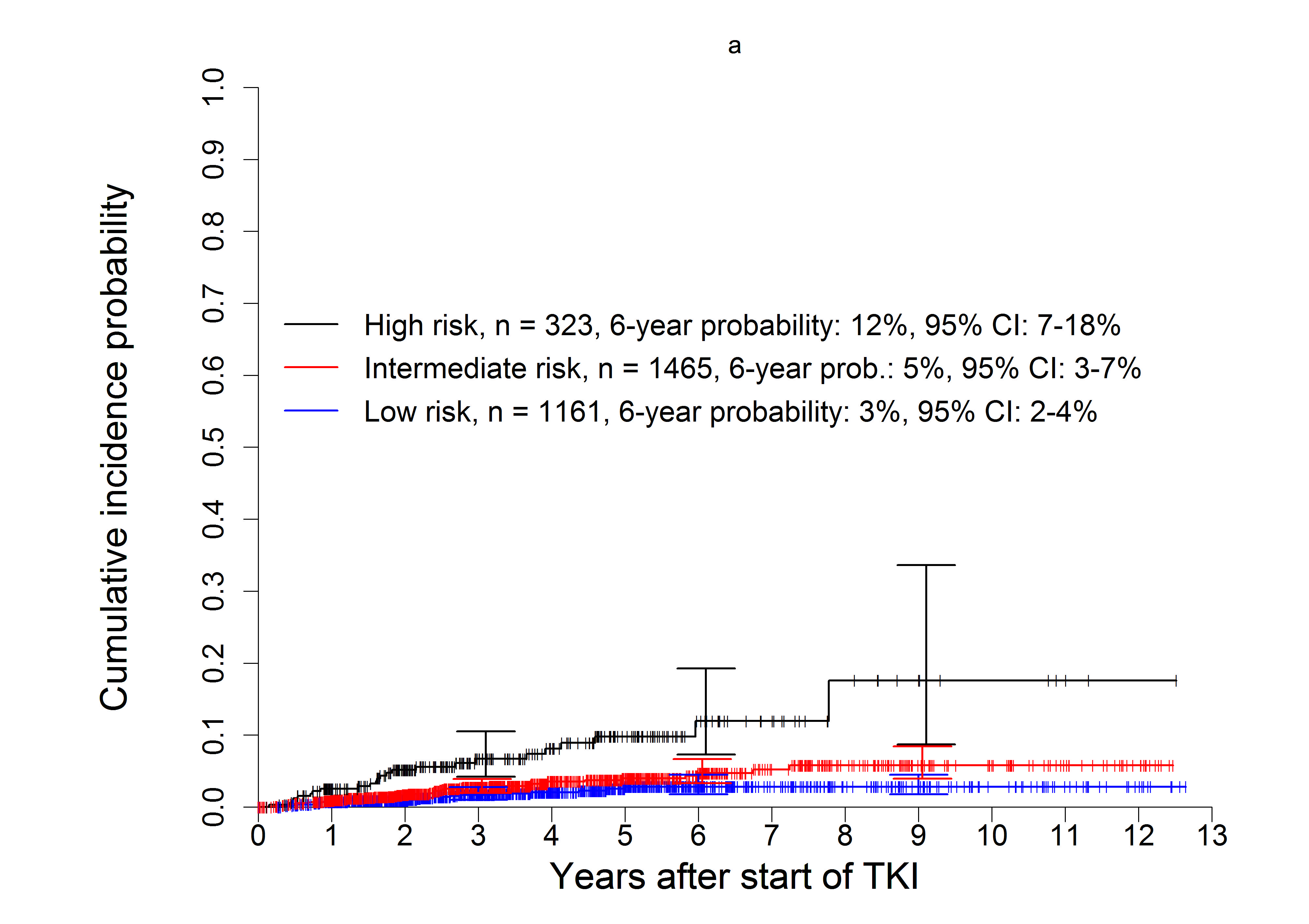


**Number of patients still at risk (n) at different years of observation**

| ***Year*** | ***0*** | ***3*** | ***6*** | ***9*** |
| --- | --- | --- | --- | --- |
| *Low risk, n* | 1161 | 631 | 201 | 58 |
| *Intermediate risk, n* | 1465 | 761 | 223 | 59 |
| *High risk, n* | 323 | 147 | 36 | 9 |

At 3, 6, and 9 years, horizontal crossbars indicate the upper and lower limit of the 95% confidence interval (CI) for the estimated probability. The high-risk group of the Euro score, though not the intermediate-risk group, had signiﬁcantly higher probabilities of dying because of CML than the low-risk group, P < 0.0001 and P = 0.0531 respectively. The corresponding hazard ratios were 4.368 (95% CI: 2.430–7.852) and 1.670 (95% CI: 0.993–2.808). The concordance indices at 1, 5, and 10 years were 60.6, 60.5, and 62.4, respectively.

**b) stratified for the risk groups according to the EUTOS score**


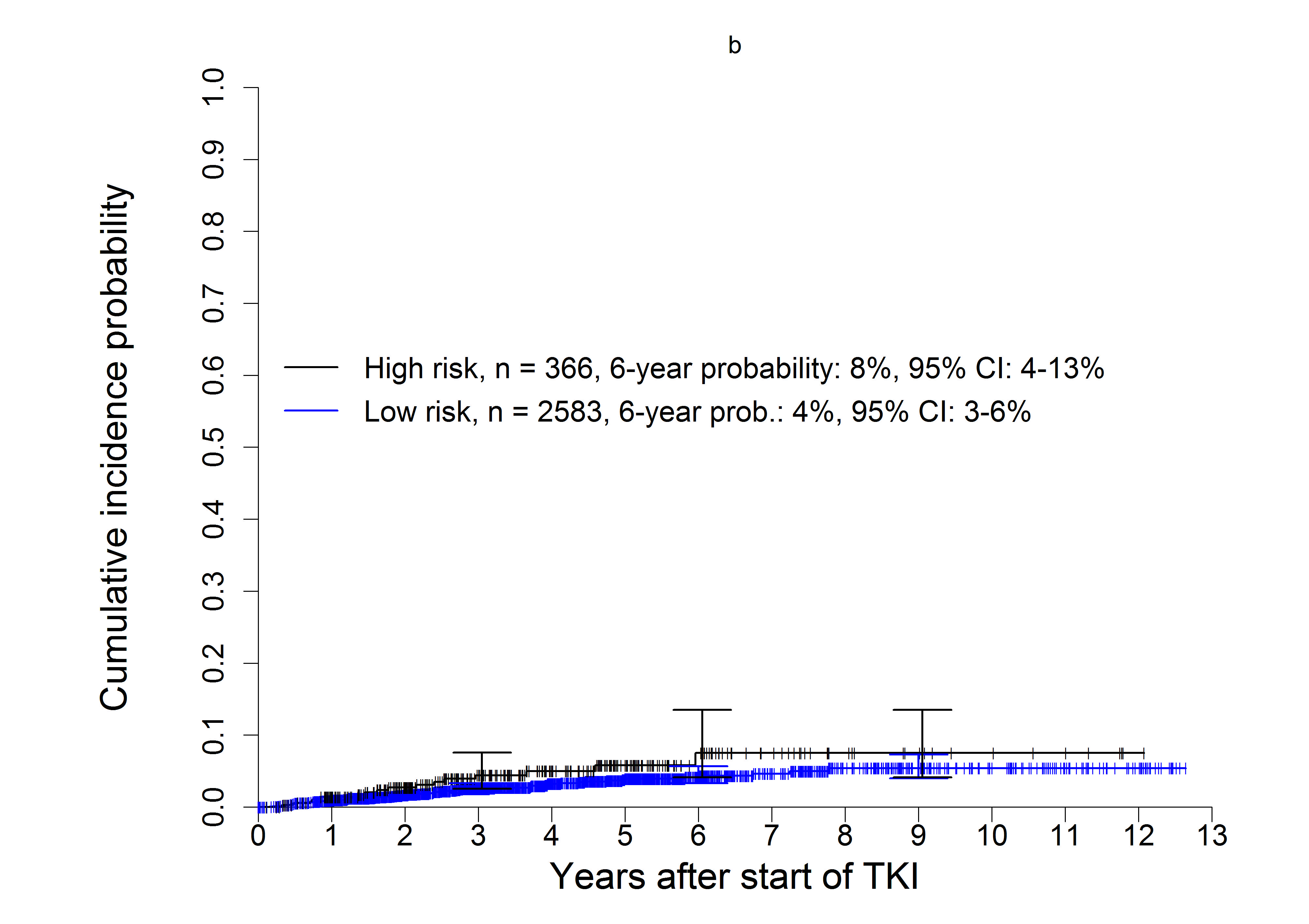


**Number of patients still at risk (n) at different years of observation**

| ***Year*** | ***0*** | ***3*** | ***6*** | ***9*** |
| --- | --- | --- | --- | --- |
| *Low risk, n* | 2583 | 1351 | 410 | 113 |
| *High risk, n* | 366 | 188 | 50 | 13 |

At 3, 6, and 9 years, horizontal crossbars indicate the upper and lower limit of the 95% confidence interval (CI) for the estimated probability. The probabilities of dying because of CML were not signiﬁcantly different between the two risk groups, P = 0.0964. The corresponding hazard ratio was 1.582 (95% CI: 0.921–2.716). The concordance indices at 1, 5, and 10 years were 52.7, 52.4, and 52.4, respectively.

**Figure 3. State occupation probabilities for death with and without progression according to competing risk and progressive illness-death model in 2949 patients from the combined out-study and population-based registry sections**

**
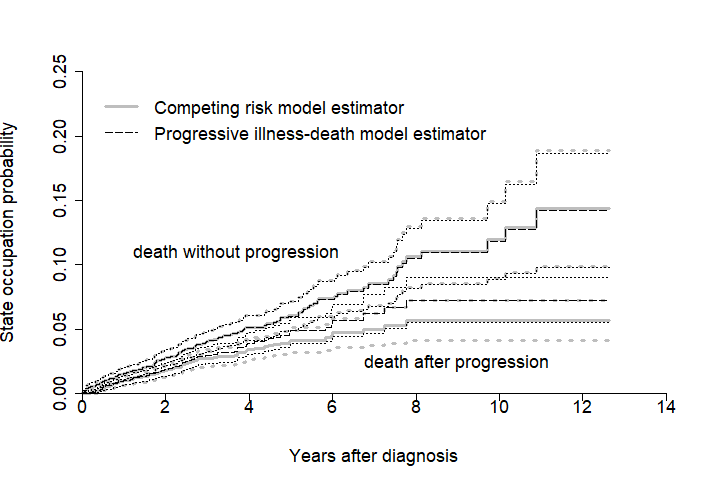
**

After 8 years, the probability of death after progression was 7.3% with the progressive illness-death model and 5.7% with the competing risk model. In contrast, regarding death without progression, differences were small. At 8 years, the corresponding probabilities amounted to 10.5% and 10.6%.

**Figure 4. Overall survival probabilities in 2949 patients from the combined out-study and population-based registry sections**

**a) stratified for the risk groups according to the Euro score**


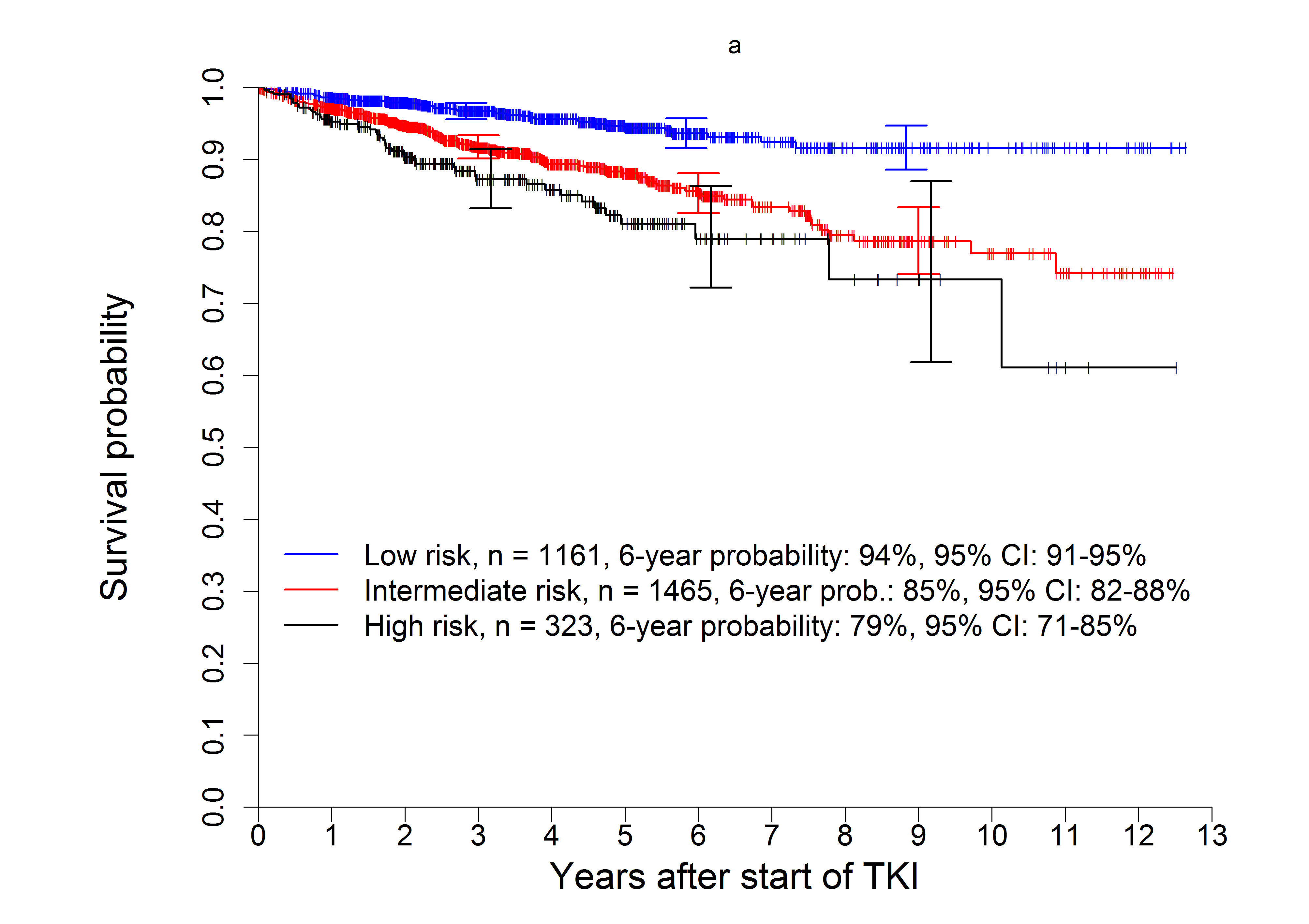


**Number of patients still at risk (n) at different years of observation**

| ***Year*** | ***0*** | ***3*** | ***6*** | ***9*** |
| --- | --- | --- | --- | --- |
| *Low risk, n* | 1161 | 631 | 201 | 58 |
| *Intermediate risk, n* | 1465 | 761 | 223 | 59 |
| *High risk, n* | 323 | 147 | 36 | 9 |

At 3, 6, and 9 years, horizontal crossbars indicate the upper and lower limit of the 95% confidence interval (CI) for the estimated probability. The intermediate- and high-risk groups of the Euro score had signiﬁcantly lower survival probabilities than the low-risk group with both P < 0.0001. The corresponding hazard ratios were 2.472 (95% CI: 1.783–3.427) and 3.687 (95% CI: 2.448–5.553). The concordance indices at 1, 5, and 10 years were 61.1, 61.3, and 61.4, respectively.

**b) stratified for the risk groups according to the EUTOS score**


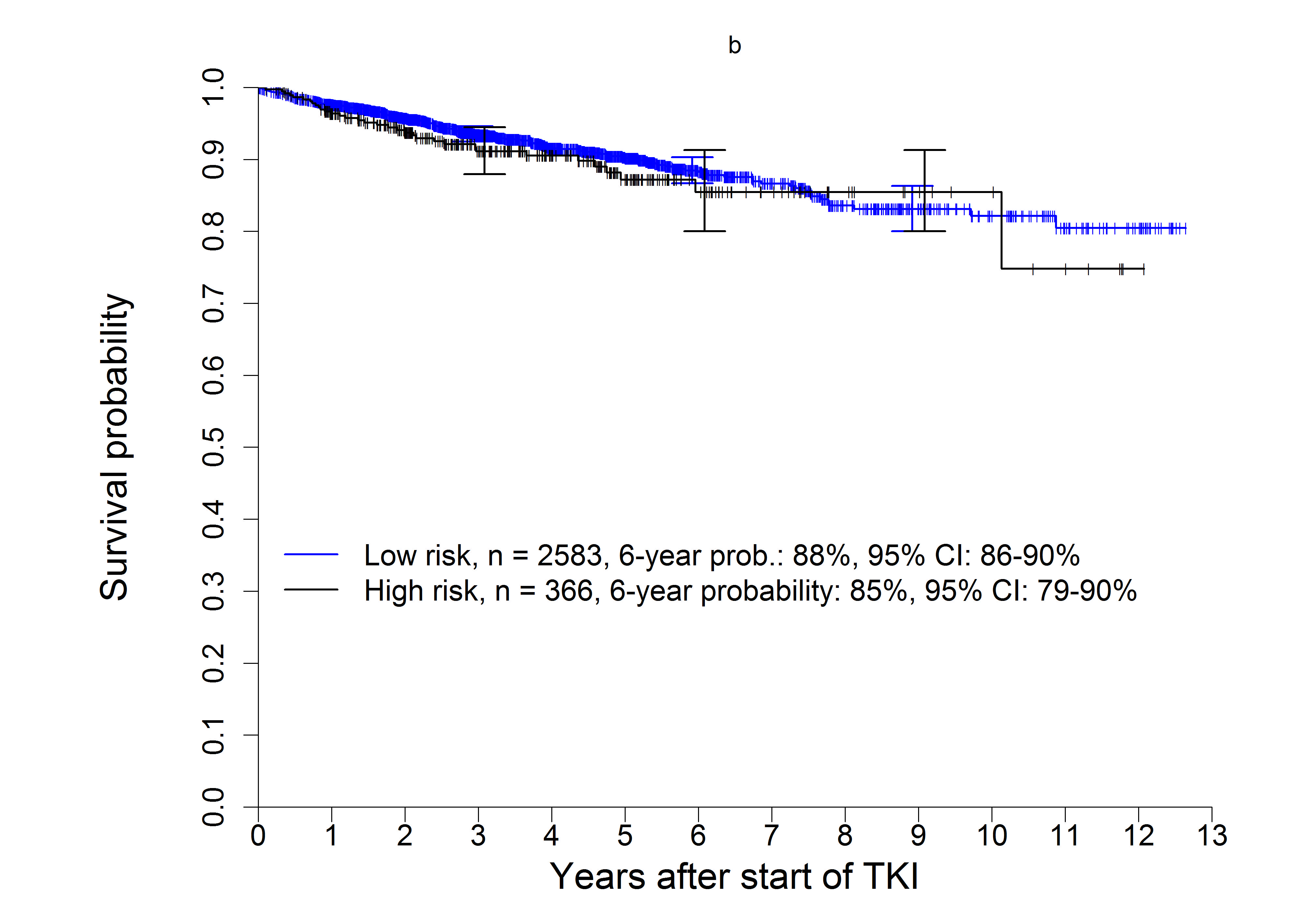


**Number of patients still at risk (n) at different years of observation**

| ***Year*** | ***0*** | ***3*** | ***6*** | ***9*** |
| --- | --- | --- | --- | --- |
| *Low risk, n* | 2583 | 1351 | 410 | 113 |
| *High risk, n* | 366 | 188 | 50 | 13 |

At 3, 6, and 9 years, horizontal crossbars indicate the upper and lower limit of the 95% confidence interval (CI) for the estimated probability. The survival probabilities were not signiﬁcantly different between the two risk groups, P = 0.2587. The corresponding hazard ratio was 1.233 (95% CI: 0.857–1.773). The concordance indices at 1, 5, and 10 years were 50.8, 50.4, and 49.9, respectively.

**Figure 5. Cumulative incidence probabilities of dying because of CML in 5154 patients from all three combined registry sections**

**a) stratified for the risk groups according to the Euro score**


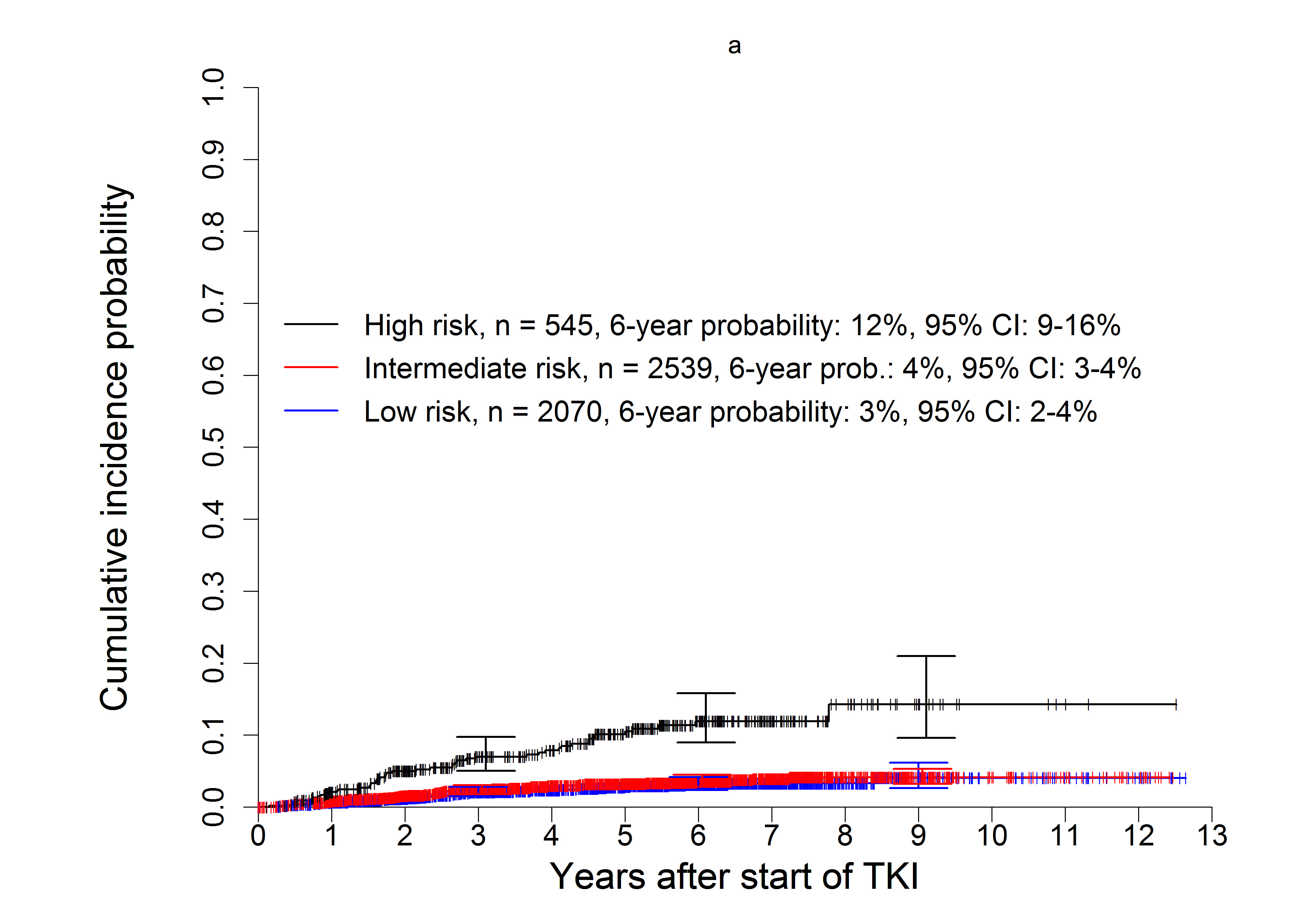


**Number of patients still at risk (n) at different years of observation**

| ***Year*** | ***0*** | ***3*** | ***6*** | ***9*** |
| --- | --- | --- | --- | --- |
| *Low risk, n* | 2070 | 1451 | 731 | 75 |
| *Intermediate risk, n* | 2539 | 1745 | 814 | 94 |
| *High risk, n* | 545 | 332 | 137 | 16 |

At 3, 6, and 9 years, horizontal crossbars indicate the upper and lower limit of the 95% confidence interval (CI) for the estimated probability. Not the intermediate- but the high-risk group of the Euro score had signiﬁcantly higher probabilities of dying because of CML than the low-risk group, P = 0.3768 and P < 0.0001, respectively. The corresponding hazard ratios were 1.173 (95% CI: 0.823–1.672) and 3.787 (95% CI: 2.564–5.595). The concordance indices at 1, 5, and 10 years were 58.0, 59.7, and 60.1, respectively.

**b) stratified for the risk groups according to the EUTOS score**


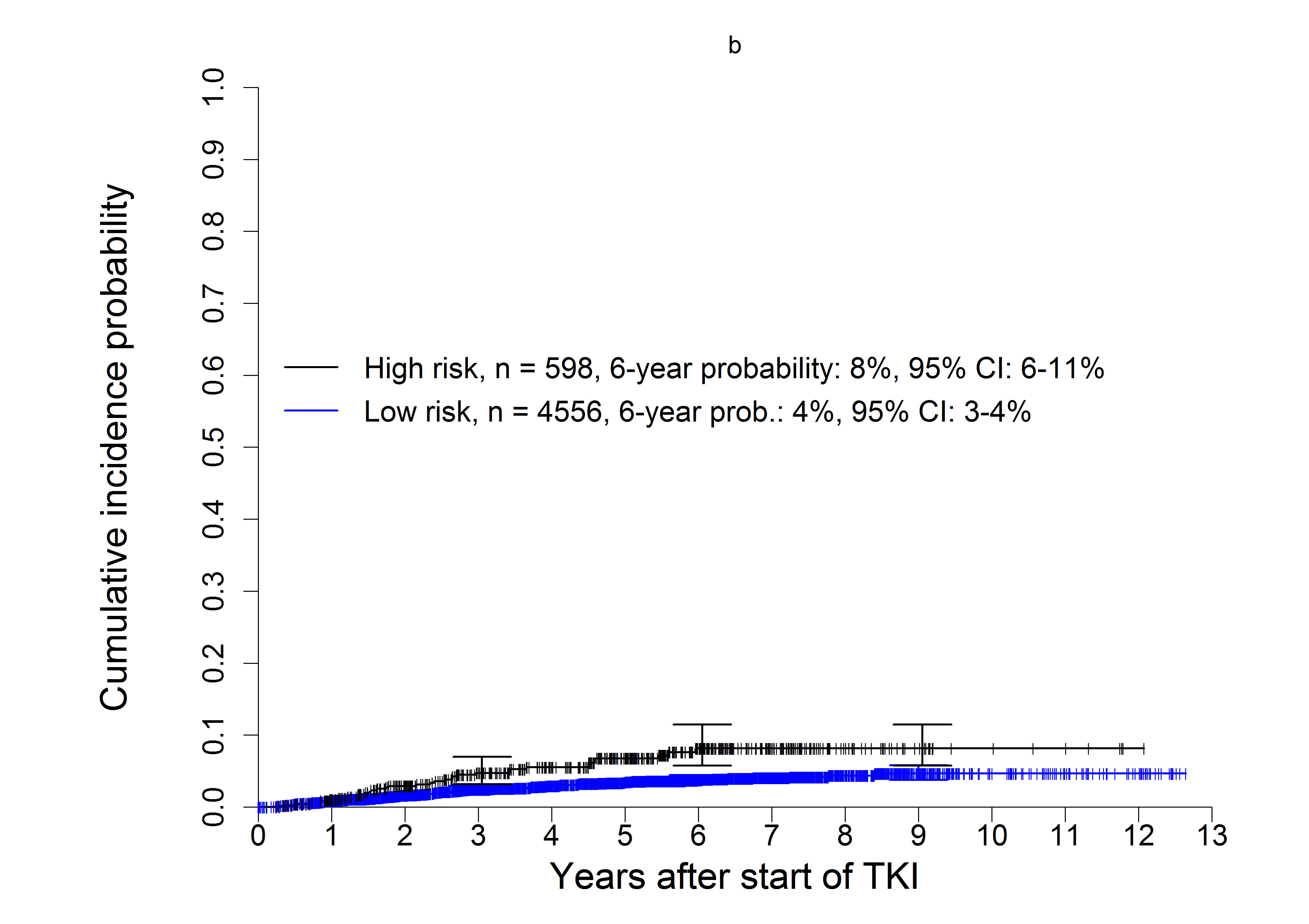


**Number of patients still at risk (n) at different years of observation**

| ***Year*** | ***0*** | ***3*** | ***6*** | ***9*** |
| --- | --- | --- | --- | --- |
| *Low risk, n* | 4556 | 3146 | 1519 | 166 |
| *High risk, n* | 598 | 382 | 163 | 19 |

At 3, 6, and 9 years, horizontal crossbars indicate the upper and lower limit of the 95% confidence interval (CI) for the estimated probability. The high-risk group of the EUTOS score had signiﬁcantly higher probabilities of dying because of CML than the low-risk group, P = 0.0002. The corresponding hazard ratio was 2.023 (95% CI: 1.393–2.940). The concordance indices at 1, 5, and 10 years were 50.8, 54.4, and 54.2, respectively.

**Figure 6. State occupation probabilities for death with and without progression according to competing risk and progressive illness-death model in 5154 patients from all three combined registry sections**


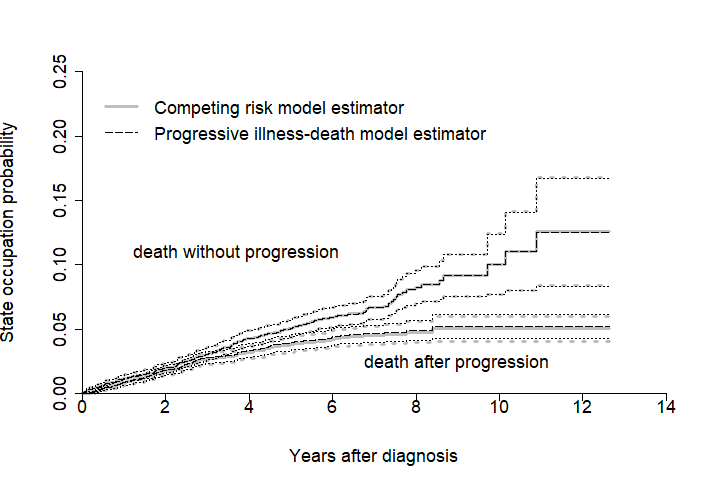


Differences in the state occupation probabilities were small for either kind of death. After 8 years, the probability of death after progression was 4.9% with the progressive illness-death model and 4.8% with the competing risk model. Regarding death without progression, the corresponding probabilities both amounted to 8.3%.

**Figure 7. Overall survival probabilities in 5154 patients from the combined in-study, out-study, and population-based registry sections**

**a) stratified for the risk groups according to the Euro score**


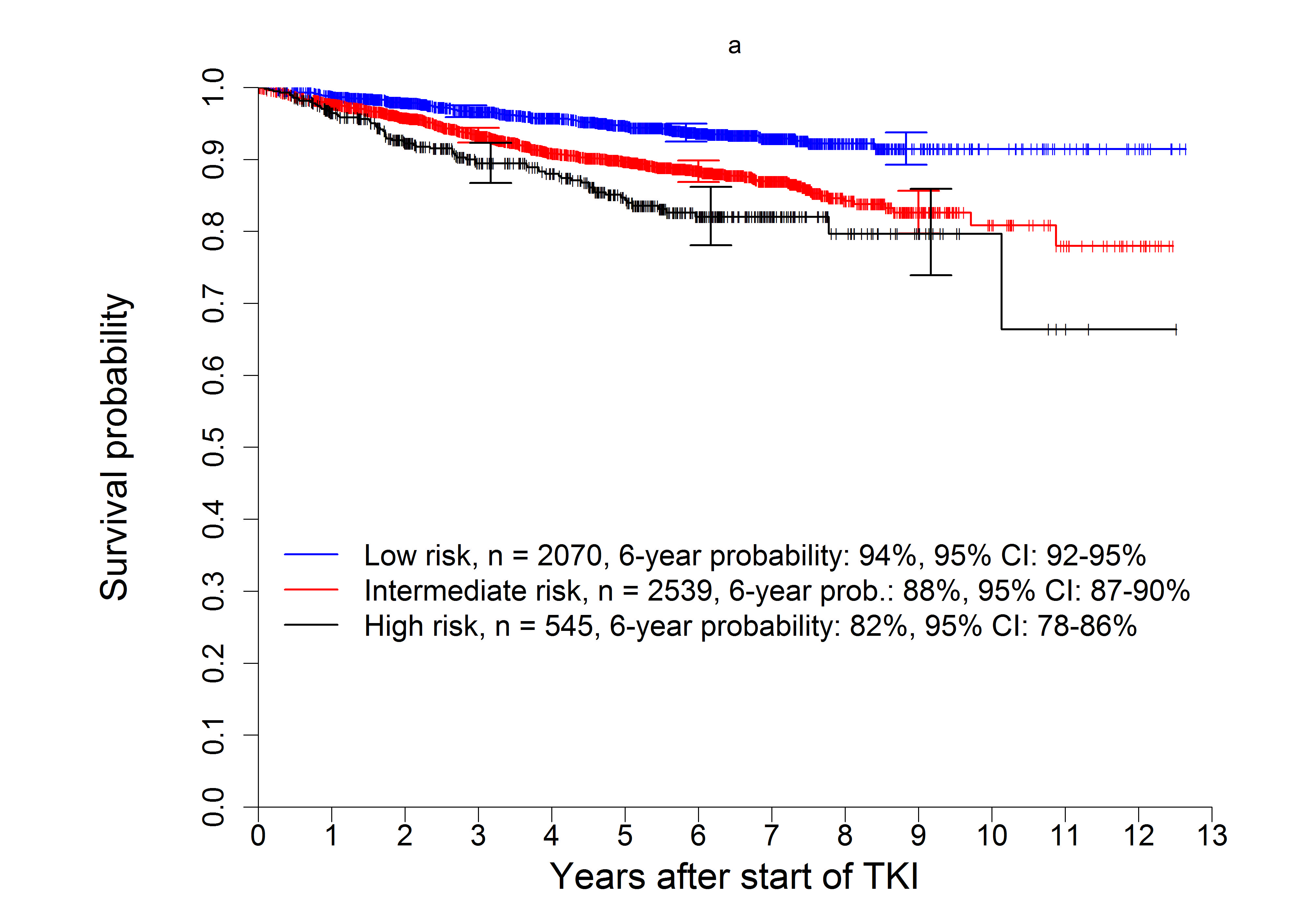


**Number of patients still at risk (n) at different years of observation**

| ***Year*** | ***0*** | ***3*** | ***6*** | ***9*** |
| --- | --- | --- | --- | --- |
| *Low risk, n* | 2070 | 1451 | 731 | 75 |
| *Intermediate risk, n* | 2539 | 1745 | 814 | 94 |
| *High risk, n* | 545 | 332 | 137 | 16 |

At 3, 6, and 9 years, horizontal crossbars indicate the upper and lower limit of the 95% confidence interval (CI) for the estimated probability. The intermediate- and high-risk groups of the Euro score had signiﬁcantly lower survival probabilities than the low-risk group with both P < 0.0001. The corresponding hazard ratios were 2.004 (95% CI: 1.596–2.517) and 2.925 (95% CI: 2.169–3.943). The concordance indices at 1, 5, and 10 years were 59.3, 59.6, and 59.4, respectively. **b) stratified for the risk groups according to the EUTOS score**


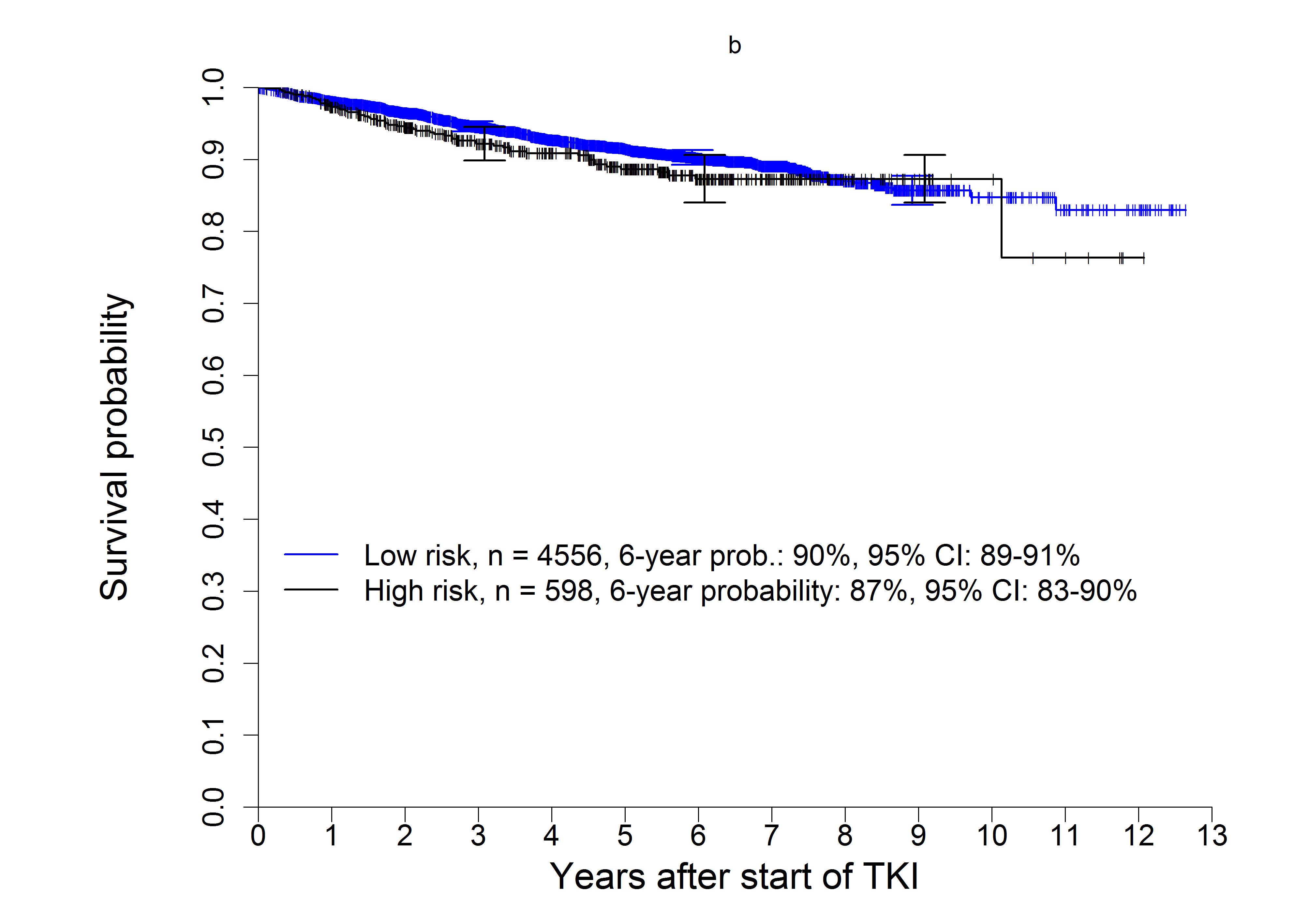


**Number of patients still at risk (n) at different years of observation**

| ***Year*** | ***0*** | ***3*** | ***6*** | ***9*** |
| --- | --- | --- | --- | --- |
| *Low risk, n* | 4556 | 3146 | 1519 | 166 |
| *High risk, n* | 598 | 382 | 163 | 19 |

At 3, 6, and 9 years, horizontal crossbars indicate the upper and lower limit of the 95% confidence interval (CI) for the estimated probability. The survival probabilities of the high-risk group were not signiﬁcantly different from the ones of the low-risk group, P = 0.0739. The corresponding hazard ratio was 1.290 (95% CI: 0.976–1.704). The concordance indices at 1, 5, and 10 years were 51.1, 51.4, and 50.7, respectively.
